# Clinical and historical infection of Tacheng tick virus 2: A retrospective investigation

**DOI:** 10.1371/journal.pntd.0012168

**Published:** 2024-06-13

**Authors:** Yuqing Jia, Yu Zhang, Xuanchen Wu, Zhihui Dong, Songsong Xie, Wei Li, Quan Liu, Xiaobo Lu, Yuanzhi Wang

**Affiliations:** 1 The People’s Hospital of Xixian, Xinyang, China; 2 Department of Basic Medicine, School of Medicine, Shihezi University, Shihezi, China; 3 Key Laboratory for Prevention and Control of Emerging Infectious Diseases and Public Health Security, the Xinjiang Production and Construction Corps, Shihezi University, Shihezi, China; 4 The Sixth Division Hospital of Xinjiang Production and Construction Corps, Wujiaqu, China; 5 First Affiliated Hospital of Shihezi University, Shihezi, China; 6 Department of Basic Medicine, School of Medicine, Tarim University, Aral, China; 7 School of Life Sciences and Engineering, Foshan University, Guangdong, China; 8 The First Hospital Xinjiang Medical University, Urmqi, China; University of Pittsburgh School of Medicine, UNITED STATES

## Abstract

**Background:**

Tacheng tick virus 2 (TcTV-2) is an emerging tick-borne virus belonging to the genus Uukuvirus in the family Phenuiviridae. Initially isolated in 2019 from a patient in Xinjiang Uygur Autonomous Region (XUAR), northwestern China, who developed fever and headache after a tick bite, TcTV-2 was concurrently molecularly detected in hard ticks across various countries, including China, Kazakhstan, Romania, and Turkey. This study conducted a retrospective epidemiological investigation of TcTV-2 infection.

**Methodology:**

In this retrospective cohort study, we collected samples from 47 tick-bitten patients, 984 herdsmen, 7 Asian badgers, 13 red foxes, and 168 *Hyalomma asiaticum* tick egg batches. Patients’ samples were primarily analyzed by using high-throughput sequencing, targeting the V3-V4 region of the bacterial 16S rRNA gene and viral cDNA libraries. Typical tick-borne pathogens were further confirmed using RT-PCR and detected in Asian badgers, red foxes and *Hy*. *asiaticum* tick egg batches. We also conducted enzyme-linked immunosorbent assay (ELISA) to detected specific IgM and IgG antibodies against TcTV-2 in herdsmen. Phylogenetic analysis was performed to genetically characterize TcTV-2 detected in this study.

**Principal findings:**

TcTV-2 was detected in various samples, including blood, urine, and throat swabs from 12.77% (6/47) tick-bitten patients. It was found in blood samples of 14.29% (1/7) of wild badgers, 7.69% (1/13) of red foxes, and 13.69% (23/168) of *Hy*. *asiaticum* egg batches. Furthermore, ELISA results revealed that 9.55% (94/984) of the serum samples (34 from males and 60 from females) were tested positive for TcTV-2-specific IgG, while 2.95% (29/984, 7 males and 22 females) showed positivity for TcTV-2-specific IgM. Additionally, 1.02% (10/984, 4 males and 6 females) of the sera tested positive for both TcTV-2-specific IgM and IgG. Phylogenetic analysis indicated that the TcTV-2 strains detected in this study were genetically similar, regardless of their origin and host species.

**Conclusions:**

Clinical symptoms of TcTV-2 infection in patients are nonspecific, with common symptoms including headache, fever, asthenia, vomiting, myalgia, rash, and meningitis-like signs. TcTV-2 can be detected in blood, urine, and throat swab samples of infected patients. Among local herdsmen, 9.55% tested positive for TcTV-2-specific IgG and 2.95% for TcTV-2-specific IgM. Importantly, TcTV-2 can be transovarially transmitted in *Hy*. *asiaticum* ticks, and the Asian badgers and red foxes are potential reservoirs of TcTV-2.

## Author summary

Tacheng tick virus 2 was initially identified in China through a metagenomic analysis of ticks. A patient bitten by a tick was subsequently found to be infected with the virus, suggesting an association with human febrile disease. Subsequent detections of TcTV-2 in various tick species have been reported in Kazakhstan, Romania, Turkey, Poland, Ukraine, and Georgia. However, the infection dynamics of this emerging tick-borne virus in humans and animals remain unclear. In this study, we identified 6 additional patients infected with TcTV-2, presenting with nonspecific clinical symptoms such as fever, asthenia, vomiting, myalgia, rash, and signs resembling meningitis. TcTV-2 was detectable in blood, urine, and throat swabs. Moreover, 9.55% of local herdsmen tested positive for TcTV-2-specific IgG and 2.95% tested positive for specific IgM. We also identified Asian badgers and red foxes as potential animal reservoirs, highlighting that TcTV-2 can be transovarially transmitted in *Hyalomma asiaticum* ticks. These findings significantly contribute to our understanding of the infection and transmission dynamics of this emerging tick-borne virus.

## Introduction

The Phenuiviridae family encompasses more than 70 virus species [1–3]. Recent years have witnessed the emergence of various tick-borne bunyaviruses, including Tacheng tick virus 1, Guertu virus, and Tamdy virus [4,5], leading to human infections in Xinjiang Uygur Autonomous Region (XUAR), northwestern China [6].

In 2019, another virus within the family Phenuiviridae, Tacheng tick virus 2 (TcTV-2), was first isolated from a tick-bitten patient in XUAR, presenting symptoms of fever and headache [7]. Simultaneously, TcTV-2 was molecularly detected in hard ticks across multiple countries, including China, Kazakhstan, Romania, and Turkey [8–10]. Despite these findings, systematic information regarding clinical symptoms, natural reservoirs, transovarial transmission of ticks, serological positivity in herdsmen to TcTV-2 infection remains unclear. To address this, we conducted a retrospective epidemiological study of TcTV-2 infection in northwestern China, involving 47 hospitalized tick-bitten patients, 20 wildlife (comprising 7 Asian badgers and 13 red foxes), 168 *Hyalomma asiaticum* tick egg batches, and 984 herdsmen.

## Materials and methods

### Ethics statement

This study was reviewed and approved by the ethics committee of School of Medicine, Shihezi University in accordance with the medical regulations of China (Approval numbers A2015-063-01 and KJ2020-062-01). All participants provided written informed consent. Furthermore, all the protocols, experimentation, and animal manipulation were thoroughly reviewed and approved by the Animal Welfare Committee of Shihezi University (Approval numbers A2018-144-01 and A2020-113-01).

### Study area and sample collection

In this retrospective cohort study, we collected eligible-hospitalized patients with a history of fever, headache, and tick bites from 5 sentinel hospitals in XUAR, spanning the years 2017 to 2021. We used a standardized surveillance form to collect the clinical and epidemiological data, which included demographic information, underlying medical conditions, recent tick exposures, clinical symptoms, laboratory test results, and treatment details. We collected blood, urine, throat swab, and cerebrospinal fluid (CSF) specimens from 47 tick-bitten patients across 4 sites in 5 years (Shihezi, Manas, Shawan, and Qinghe; from 2017 to 2021). Medical records were also obtained for these patients. All patient samples were subjected to reverse transcription polymerase chain reaction (RT-PCR) analysis, and patients with positive results were considered infected with TcTV-2.

In addition, we gathered road-killed or illegally hunted wildlife, including 7 Asian badgers and 13 red foxes, in Nilka County, XUAR. Furthermore, 168 *Hy*. *asiaticum* tick egg batches were collected from engorged female ticks on pastured Bactrian camels (*Camelus bactrianus*).

### Sample treatment and detection

We extracted total RNA and DNA from the collected samples using the FastPure Cell/Tissue Total RNA Isolation Kit (Vazyme, China) and DNA Extraction Kit (Tiangen, China), respectively. Then, the RNA was reverse-transcribed into cDNA using the Reverse Transcription Kit (TransGen Biotech, China). Subsequently, all samples underwent partial S segment detection through nested RT-PCR using the specific primers (S1 and S2 Tables) [7]. The PCR products were sequenced to confirm the positive results. For patients’ samples, we conducted high-throughput sequencing, focusing on the V3-V4 region of the bacterial 16S rRNA gene and viral cDNA libraries. The analysis was performed by Huajin Biotech Co. in Shanghai, China. Typical tick-borne pathogens were further validated using PCR or RT-PCR methods (S1 and S2 Tables). The positive amplicons were sequenced by Huajin Biotech Co. in Shanghai, China.

### Sequence analysis

We compared all sequences obtained in this study in GenBank available using the nucleotide BLAST program (http://www.ncbi.nlm.nih.gov/BLAST/) and subsequently deposited these sequences in the GenBank database (https://www.ncbi.nlm.nih.gov/nuccore/?term=, S4 Table). To establish phylogenetic relationships, we employed the software package Mega VII, utilizing the maximum likelihood method. Bootstrap values were calculated based on 1,000 replicates [11].

### Serologic tests

We collected a total of 984 herdsmen from 5 animal husbandry villages of Manas County (S3 Table). These samples were obtained as part of routine physical examinations conducted at the local Centers for Disease Control and Prevention. To investigate specific immunoglobulin M (IgM) and IgG antibodies against TcTV-2, we inserted the entire nucleocapsid protein encoding gene (S segment), referred to as TcTV-2-N gene, into the expression vector pET-30a. This construct was expressed in *Escherichia coli* BL21 (DE3) strain. Subsequently, the TcTV-2-N protein was purified using affinity chromatography (Ni-IDA resin) and the purity of the TcTV-2-N protein was confirmed by western blot (S1 Fig). For the detection of antibodies, we developed an indirect enzyme-linked immunosorbent assay (ELISA), using the purified TcTV-2-N as a coating protein (details of ELISA in S3 Table). This assay was employed to analyze the samples from the 984 local herdsmen, and the results were analyzed using IBM SPSS Statistics.

## Results

A total of 47 tick-bitten patients who presented to the hospital had a history of tick bites. Among them, 6 patients were confirmed to have TcTV-2 infection as TcTV-2 RNA tested positive in their blood, urine, or throat swab samples using nested RT-PCR. Furthermore, 1 patient was further confirmed through full-length sequencing of TcTV-2 S and L segments, which have been deposited in GenBank under accession numbers MK820045 and MK801756, respectively, out of other 5 tick-bitten patients. The age range of 6 patients, consisting of 3 females and 3 males, was 29 to 82 years. Their clinical syndromes are detailed in Table 1. The most common symptoms were headache and fever, with temperatures ranging from 37.9 to 39.5°C, which were observed in 6 patients. Five of the patients experienced asthenia, while vomiting, myalgia, rash, and meningitis-like symptoms were present in 3 patients. Additional clinical findings included anorexia in 2 patients, chest distress in 2 patients, and lymphadenopathy in 1 patient (Table 1).

**Table 1:** Clinical information of the 6 patients positive for the TcTV-2 detection.

Characteristics	Patient 1	Patient 2	Patient 3	Patient 4	Patient 5	Patient 6
Gender	F	M	F	M	F	M
Age	82 yr	29 yr	38 yr	43 yr	42 yr	72 yr
Tick-bite location	head	arm	abdomen	unknown	auricle	head
Past medical history	No	No	Hepatitis C	No	No	No
Highest temperature	39.0°C	39.5°C	39.0°C	40.0°C	39.7°C	39.8°C
Asthenia	+	-	+	+	+	+
Headache	+	+	+	+	+	+
Vomiting	-	+	+	+	-	-
Anorexia	-	+	+	-	-	-
Myalgia	+	-	-	-	+	+
Arthralgia	-	-	-	-	-	+
Chest distress	-	+	+	-	-	-
Unconsciousness	-	-	+	+	-	-
Meningitis stimulus	-	+	+	+	-	-
Lymphadenopathy	+	-	-	-	-	-
Rash	-	-	+	-	+	+
Co-infection	-	-	Rickettsia slovaca	Rickettsia raoultii	-	Rickettsia conorii

In 2 patients, unconsciousness was observed, and they were found to be co-infected with *Rickettsia raoultii* (GenBank accession numbers MT237575 and MT219896) or *Rickettsia slovaca* (GenBank accession numbers MT237574 and MT219894). This co-infection was identified through sequencing 2 rickettsial genetic markers, including surface antigen (*sca*1) and outer membrane protein A (*omp*A). Additionally, a patient experiencing arthralgia was found to be co-infected with *Rickettsia conorii* (GenBank accession numbers MG190327, MG190328, and MG190329), which was determined by sequencing rickettsial ompA, citrate synthase (*glt*A), and surface cell antigen 4 *(sca*4). Phylogenetic tree was constructed to confirm the results (S2 Fig).

Cytological and biochemical analyses revealed several noteworthy findings in the 6 patients with TcTV-2 infection, which occurred between 2 to 14 days after hospitalization. These findings included elevated levels of leukocyte, C-reactive protein, and D-dimer. Furthermore, 3 of these patients exhibited increased albumin and a higher total leukocyte count in their cerebrospinal fluid (CSF). Furthermore, 3 of these patients exhibited increased albumin and a higher total leukocyte count in their CSF. In addition, the levels of alanine aminotransferase (ALT) or aspartate aminotransferase (AST) were elevated in 3 patients, with one of them having a prior history of hepatitis C. Two patients, who were co-infected with spotted fever group *Rickettsia*, showed elevated levels of creatine kinase and creatine kinase isoenzyme. Additionally, increased fibrinogen prothrombin ratio and international normalized ratio were found in 2 patients (Table 2).

**Table 2:** Laboratory data of the 6 patients positive for the TcTV-2 detection.

Characteristics	Patient 1	Patient 2	Patient 3	Patient 4	Patient 5	Patient 6
Red blood count (3.5–5.5×10^12^/L)	4.34	4.79	4.86	4.50	4.97	NA
Leukocyte count (4–10×10^9^/L)	6.38	11.62↑	19.80↑	15.40↑	5.67	16.70↑
Neutrophil count (2.04–7.5×10^9^/L)	3.67	10.82↑	15.13↑	13.29↑	4.56	15.20↑
lymphocyte count (0.8–4×10^9^/L)	1.94	0.63↓	3.40	0.69↓	0.69↓	0.89
Platelet count (100–300×10^9^/L)	241.0	267.0	329.0↑	246.0	131.00	55.0↓
hemoglobin concentration (120-160g/L)	118.0	141.0	132.0	130.0	142.0	NA
C-reactive protein (0.00–10.00mg/L)	0.50	<0.50	11.17↑	3.58	93.57↑	37.73↑
D-dimer (0–0.5μg/mL)	2.51↑	NA	5.76↑	652.00↑	2442.00↑	NA
Fibrinogen (2-4g/L)	2.10	3.29	6.12↑	6.12↑	3.92	NA
INR (0.8–1.1)	0.86	1.14↑	1.22↑	1.50↑	1.04	NA
Prothrombin ratio (0.75–1.15)	NA	1.14	1.22↑	25.00↑	NA	NA
AST (0-40U/L)	22.00	13.80	33.60	25.00	48.00↑	109.78↑
ALT (0-40U/L)	11.00	10.10	80.70↑	71.00↑	30.53	185.40↑
CK (30-170U/L)	56.00	86.00	259.00↑	722.00↑	94.80	NA
CK-MB (0-20U/L)	20.00	10.00	31.00↑	64.00↑	8.56	NA
Chloride content (95-105mmol/L)	109.00↑	101.00	105.00	97.00	103.50	NA
Potassium ion content (3.5–5.5 mmol/L)	4.38	3.63	4.66	3.11↓	0.92↓	3.46
Sodium ion content (135-145mmol/L)	141.30	139.00	136.00	134.00↓	134.45↓	124.46↓
Thrombin time 13-19s	18.3 s	16.1 s	14.00 s	33.5 s↑	12.5 s	NA
Leucocyte in CSF (10×10^6^/L)	NA	107.0↑	12.0↑	64.0↑	NA	NA
Albumin in CSF (200–400 mg/L)	NA	988.50↑	764.00↑	630.00↑	NA	NA
CT Scan of the Lungs	Bilateral middle lobe proliferative foci, localized emphysema in both lungs, aortic wall plaque formation	NA	Localized fibrosis in the middle lobe of the right lung and posterior basal segment of the left lower lobe, and small value-added nodules in the lingual segment of the upper lobe of the left lung	Thin dense shadows on both lung bottoms	NA	NA
CT scan of the head	Bilateral basal segmental lacunar infarction, demyelination, and cerebral atrophy	NA	NA	No obvious abnormality	NA	NA
Co-infection	-	-	Rickettsia slovaca	Rickettsia raoultii	-	Rickettsia conorii

NA, no data obtained.

All patients received empirical treatment with intravenous ceftriaxone sodium, ranging from 1 to 2 g per day, for a duration of 10 to 12 days. Following the confirmation of TcTV-2 infection, the patients underwent treatment with ribavirin at a dosage of 0.5 to 0.75 g per day, administered for 7 to 10 days. Among the 6 TcTV-2-infected patients, 3 co-infected with the spotted fever group *Rickettsia* (SFGR) were treated with doxycycline at 0.2 g per day for 6 days. A follow-up spanning 1 to 2 years revealed that none of the 6 TcTV-2–positive patients experienced permanent clinical complications or succumbed to the infection after their discharge from the hospital.

TcTV-2 was detected in the blood samples of wild badgers, with a prevalence of 14.29% (1 out of 7), as well as in wild red foxes, at a rate of 7.69% (1 out of 13). Additionally, TcTV-2 was found in 13.69% (23 out of 168) of *Hy*. *asiaticum* egg batches (Table 3 and S3 Fig).

**Table 3:** The information of collected samples.

Sample type	No. samples	No. positive	Locations of the positives	Date of sampling
Patients’ blood	47	6	Shihezi, Shawan, Manas, and Qinghe	2017–2021
Patients’ urine	47	6		2017–2021
Patients’ throat swab	47	6		2017–2021
Patients’ cerebrospinal fluid	47	0		2017–2021
Wild badger blood	7	1	Nilka	2017
Wild red fox spleen	13	1		2019–2021
Hyalomma asiaticum egg batches	168	23	Wusu	2020

**Fig 1:**
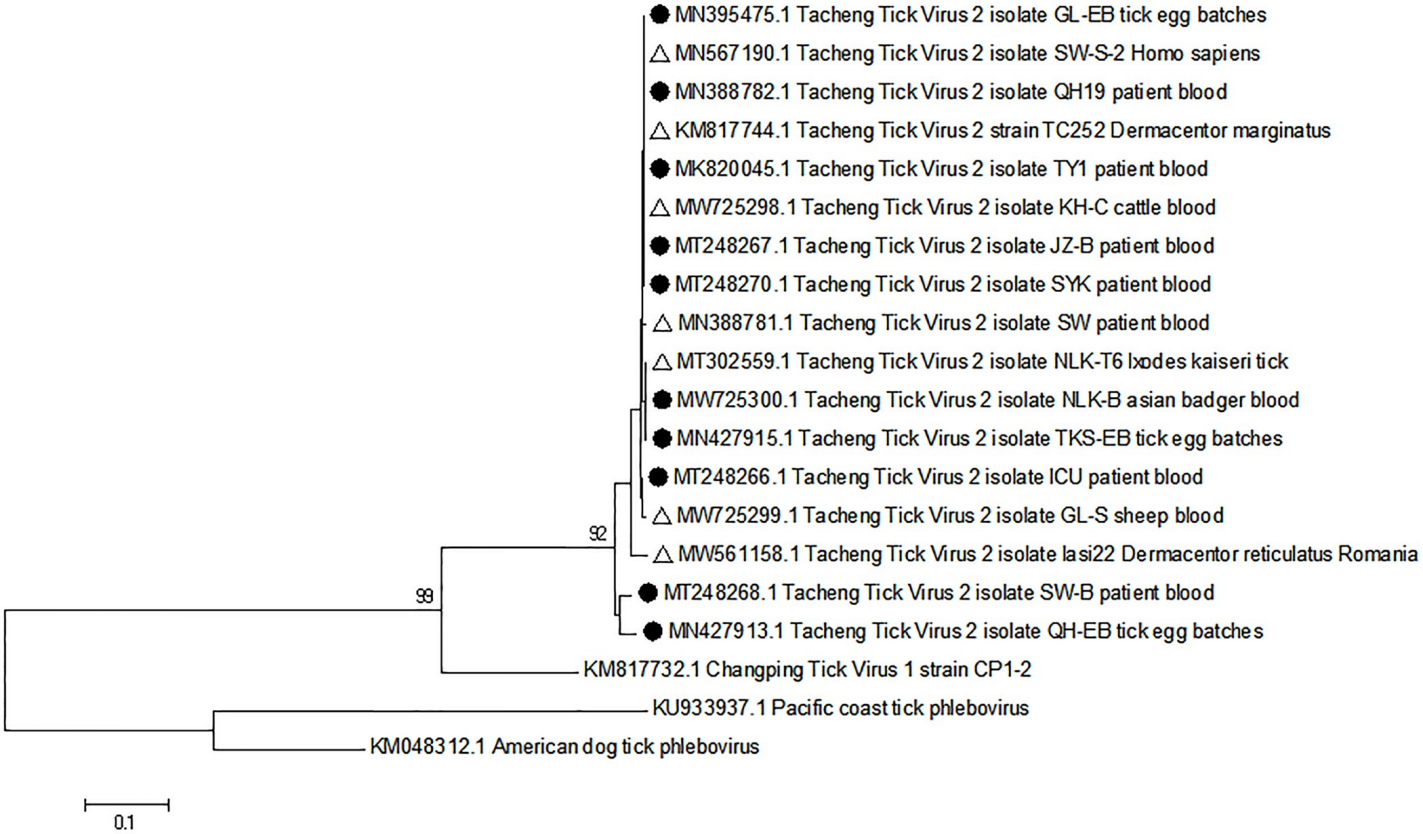
Phylogenetic relationships of the TcTV-2 in different hosts and tick species. The phylogenetic relationships were based on their partial S (365 bp–616 bp, 251 bp) segment sequences. The sequences were designated with their GenBank accession numbers and their sources. Five reference sequences (4 TcTV-2 sequences and 1 Changping tick virus 1 sequence) are marked with triangles. Samples from this study are marked with black solid dots. All the viruses were from China except the one marked with “in Romania.” Dtick, *Dermacentor marginatus*; Htick, *Hyalomma asiaticum*. (A) pET-30a-TcTV2-N was expressed in *Escherichia coli* BL21. (B) TcTV2-N was purified with Ni-IDA Affinity chromatography column. (C) The renaturated protein was confirmed by western blot assay.

Sequence alignment analysis, based on the partial TcTV-2 S segment from the 6 tick-bitten patients, revealed homologies ranging from 98.41% (248/252) to 100%. Phylogenetic analysis demonstrated that the TcTV-2 strains detected in this study exhibited a high degree of similarity among themselves, regardless of their host species or geographical origin. These TcTV-2 strains were notably distinct from Changping tick virus 1, despite sharing the same genus with TcTV-2 (Fig 1).

The ELISA results revealed that 9.55% of the serum samples (94 out of 984), consisting of 34 males and 60 females, tested positive for TcTV-2-specific IgG. Additionally, 2.95% of serum samples (29 out of 984), which included 7 males and 22 females, were positive for TcTV-2-specific IgM. Furthermore, 1.01% of the serum samples (10 out of 984), with 4 males and 6 females, were co-positive for both TcTV-2-specific IgM and IgG.

### Discussion

In a previous case involving an index patient infected with TcTV-2, clinical symptoms, such as fever, headache, localized rash, chills, severe fatigue, anorexia, nausea, vomiting, and elevated white blood cells in both blood and CSF, were reported [7]. In our retrospective study, we observed additional new clinical symptoms, including myalgia, chest distress, and lymphadenopathy. Furthermore, we noted elevated C-reactive protein, D-dimer, chloride content, and AST levels in the blood, along with lower sodium ion content. Some of these patients also displayed lesions in brain or lung CT scans. The complexity of symptoms makes it challenging for doctors to distinguish TcTV-2 infection from other arthropod-borne viruses, including members of the Phenuiviridae family.

SFGR species have been frequently identified as common pathogens in tick-bitten patients, ticks, and wild animals in XUAR [12–15]. Notably, we observed that 3 tick-bitten patients were co-infected with both TcTV-2 and SFGR, resulting in more intricate clinical symptoms, such as arthralgia and unconsciousness. This discovery highlights the challenge of distinguishing single TcTV-2 infection from cases involving multiple pathogens. As a result, it emphasizes the importance of heightened vigilance regarding co-infections of both established and emerging tick-borne pathogens in the future.

Wildlife can serve as reservoirs for various emerging arboviruses. For example, the white-tailed deer (*Odocoileus Virginianus*) has been considered a potential reservoir host for potosi virus [16]. Similarly, yellow weasels (*Mustela sibirica*) have been suggested as potential reservoirs for severe fever with thrombocytopenia syndrome virus [17]. In this study, we detected TcTV-2 in wild Asian badgers and red foxes. What’s particularly intriguing is that the wild red foxes are apex predator in the Gurbantunggut Desert, and its habitat overlaps with local residents and domestic animals. Therefore, it’s crucial to conduct further examinations on a wider range of wildlife, particularly various rodent species in this region in the future.

ELISA and PCR or RT-PCR are beneficial methods to diagnose emerging infectious diseases [18,19]. In this study, we successfully detected TcTV-2 in various samples, including blood, urine, and throat swabs from tick-bitten patients. Besides, in the future, collection of patient’s bite site swab should be added. Additionally, 9.55% (94 out of 984) of the sera from herdsmen tested positive for TcTV-2-specific IgG, while 2.95% (29 out of 984) exhibited positivity for TcTV-2-specific IgM. Notably, previous research reported the persistence of TcTV-2 nucleic acid for at least 40 days after the onset of illness in a patient’s blood sample [7]. These findings underscore the importance of exercising extra caution in areas related to blood transfusion, as well as the collection and storage of urine, sputum, or throat swab samples, given the potential presence of TcTV-2. Besides, collection of patient’s bite site swab should be added in the future.

In summary, TcTV-2 infection typically presents with nonspecific clinical symptoms, such as fever, asthenia, vomiting, myalgia, rash, and meningitis-like signs. TcTV-2 can be detected in blood, urine, and throat swab samples. In local herdsmen, 9.55% tested positive for TcTV-2-specific IgG, while 2.95% were positive for TcTV-2-specific IgM. TcTV-2 can be transovarially transmitted in *Hy*. *asiaticum* ticks, and the Asian badger and red fox are potential reservoirs of the virus.

## Supporting information

S1 Fig The expressed TcTV-2-N protein confirmed by western blotting using the anti-His antibodies. Lane M, Protein Marker; L1, TcTV-2 N protein.
(TIF)

S2 Fig Phylogenetic relationships of the *Rickettsia* detected in this study. The sequences were designated with their GenBank accession numbers and their sources. The detected sequences are marked with solid black circles.
(TIF)

S3 Fig The map of the study area. The sampling sites of tick bite patients, wildlife, and tick egg batches are respectively marked with red, green, and black solid triangles and the sampling site of herdsmen was marked with green circle. The base layer of the map was referred to https://www.usgs.gov/media/videos/avian-influenza-transmission-risk-model-web-application-virtual-tour.
(TIF)

S1 Table The primers used for RT-PCR and PCR. (DOCX)

S2 Table The reaction conditions of RT-PCR and PCR. (DOCX)

S3 Table Serological examination of the local herdsmen using ELISA. (DOCX)

S4 Table Direct link to access the accession number. (DOCX)
