# Supplementary figures and images for: Clinical and historical infection of Tacheng tick virus 2: A retrospective investigation
Source: PLoS Negl Trop Dis. 2024 Jun 13;18(6):e0012168. doi: 10.1371/journal.pntd.0012168 (PMC11175498; doi:10.1371/journal.pntd.0012168)

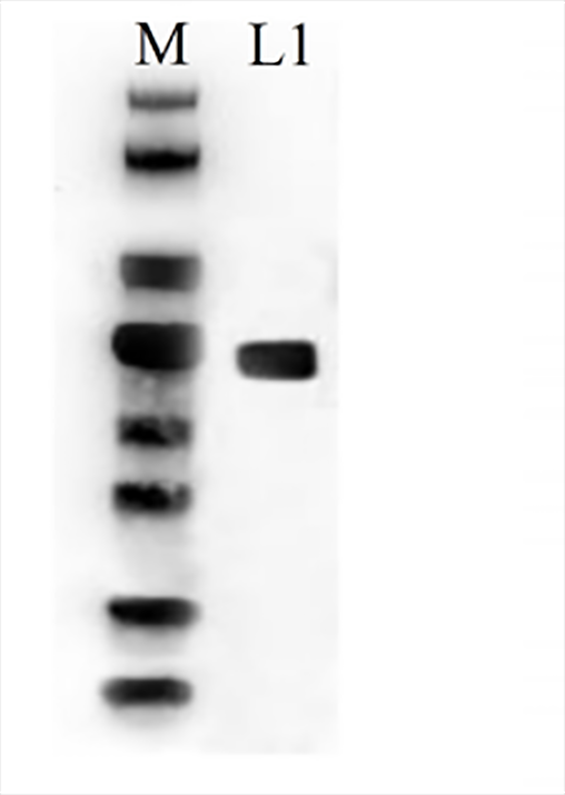

Supplement: S1 Fig — Lane M, Protein Marker; L1, TcTV-2 N protein. (TIF) [file pntd.0012168.s001.tif]

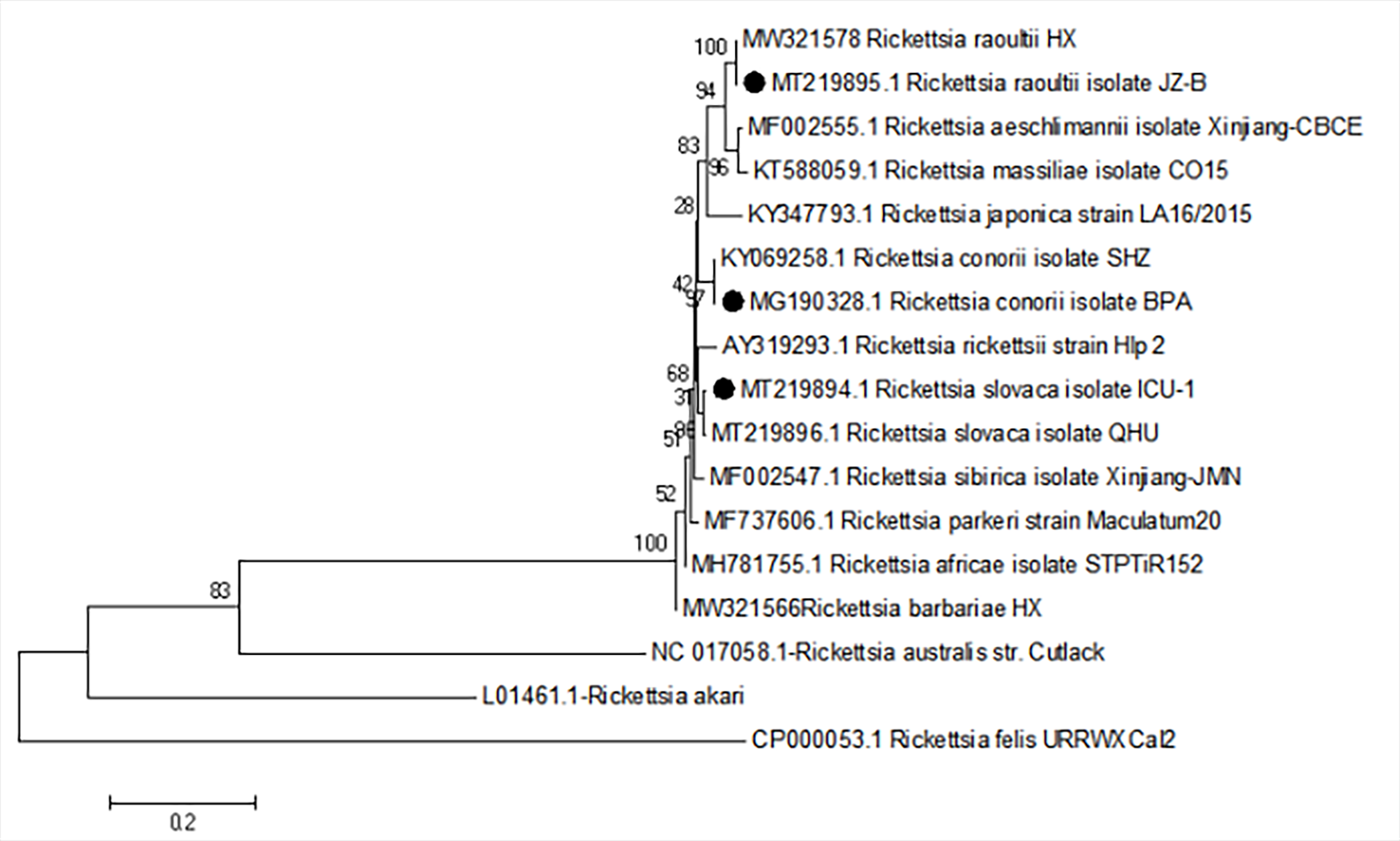

Supplement: S2 Fig — The sequences were designated with their GenBank accession numbers and their sources. The detected sequences are marked with solid black circles. (TIF) [file pntd.0012168.s002.tif]

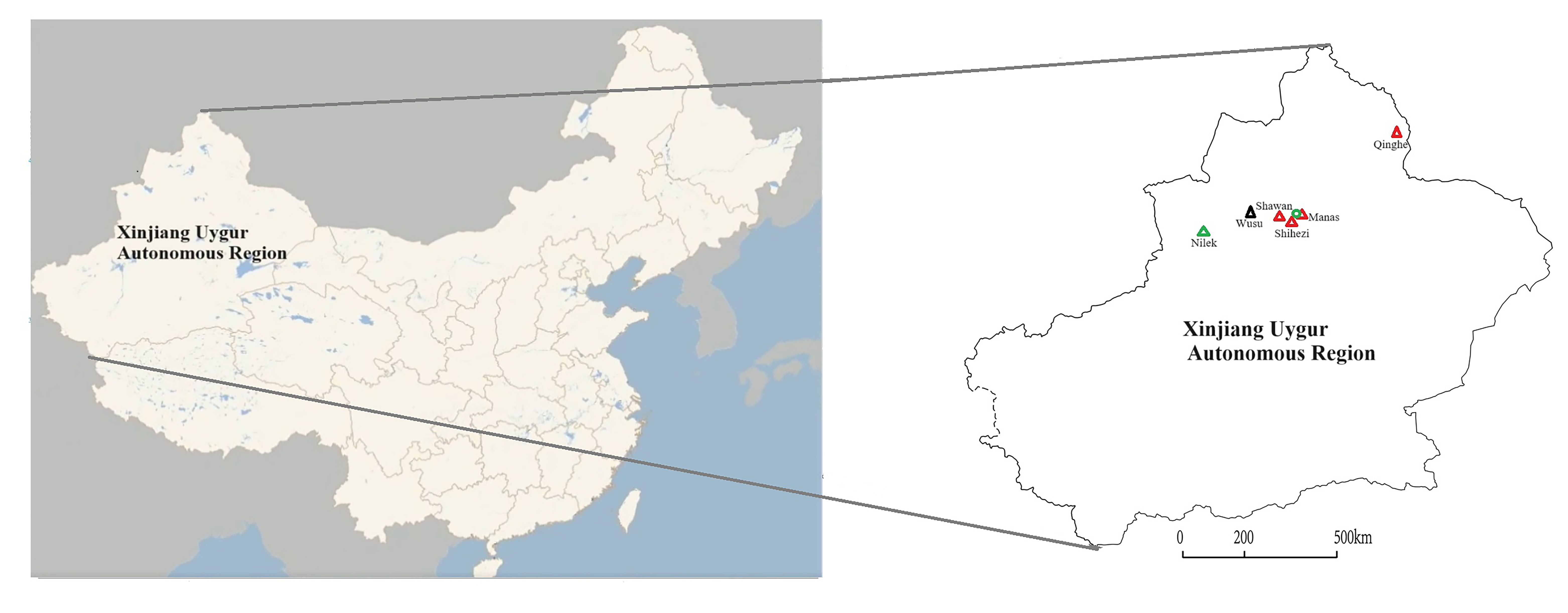

Supplement: S3 Fig — The sampling sites of tick bite patients, wildlife, and tick egg batches are respectively marked with red, green, and black solid triangles and the sampling site of herdsmen was marked with green circle. The base layer of the map was referred to https://www.usgs.gov/media/videos/avian-influenza-transmission-risk-model-web-application-virtual-tour. (TIF) [file pntd.0012168.s003.tif]
