# Supplementary material for: Clinical and historical infection of Tacheng tick virus 2: A retrospective investigation
Source: PLoS Negl Trop Dis. 2024 Jun 13;18(6):e0012168. doi: 10.1371/journal.pntd.0012168 (PMC11175498; doi:10.1371/journal.pntd.0012168)
Supplement: S1 Table — (DOCX) [file pntd.0012168.s004.docx]

**Table S1**. The primers used for RT-PCR and PCR.

| Pathogens | Targeting genes | Primer name | Primer sequence (5′→3′) |
| --- | --- | --- | --- |
| Tacheng tick virus 2 | S segment | F1 | ATCTCCTCAACGGCAACTAT |
|  |  | R1 | GACATGCGGTTCTTCATTTT |
|  |  | F2 | TCAACGGCAACTATGAGGAT |
|  |  | R2 | CTGGCTTGTATTGGAAGGA |
| Spotted fever *Rickettsia* | *ompA* | F1 | ATGGCGAATATTTCTCCAAAA |
|  |  | R1 | AGTGCAGCATTCGCTCCCCCT |
|  |  | F2 | CTTAAAGCCGCTTTATTCACCACCTC |
|  |  | R2 | CCTGTATAATTATCGGCAGGAGC |
|  | *sca1* | F1 | GGTGATGAAGAAGAGTCTC |
|  |  | R1 | CTCTTTAAAATTATGTTCTAC |
|  |  | F2 | GAGGTTTGTGGATGCGTGGT |
|  |  | R2 | ACTGTGACTTTAGTACCGACA |
|  | *gltA* | F1 | ATGACCAATGAAAATAATAAT |
|  |  | R1 | ATTGCAAAAAGTACAGTGAACA |
|  |  | F2 | GGAATCTTGCGGCATCGAGGATATG |
|  |  | R2 | CCATAGCTTTATAGATAATACCCG |
