# Supplementary material for: Clinical and historical infection of Tacheng tick virus 2: A retrospective investigation
Source: PLoS Negl Trop Dis. 2024 Jun 13;18(6):e0012168. doi: 10.1371/journal.pntd.0012168 (PMC11175498; doi:10.1371/journal.pntd.0012168)
Supplement: S2 Table — (DOCX) [file pntd.0012168.s005.docx]

**Table S2**. The reaction conditions of RT-PCR and PCR.

| **Pathogens** | **Targeting genes** | **Primers** | **RT-PCR or PCR reaction conditions** |
| --- | --- | --- | --- |
| Tacheng tick virus 2 | S segment | F1→R1 | An initial denaturation for 5 min at 94°C followed by 35 cycles of denaturation at 94℃ for 30 s, annealing at 53℃ for 30 s, and extension at 72℃ for 30 s, with a final extension at 72°C for 6 min. |
|  |  | F2→R2 | An initial denaturation for 5 min at 94°C, followed by 35 cycles of denaturation at 94℃ for 30 s, annealing at 55℃ for 30 s, and extension at 72℃ for 20 s, with a final extension at 72°C for 6 min. |
| Spotted fever *Rickettsia* | *ompA* | F1→R1 | An initial denaturation for 5 min at 95°C followed by 35 cycles of denaturation at 95℃ for 30 s, annealing at 50℃ for 30 s, and extension at 72℃ for 30 s, with a final extension at 72°C for 6 min. |
|  |  | F2→R2 | An initial denaturation for 5 min at 95°C followed by 35 cycles of denaturation at 95℃ for 30 s, annealing at 59℃ for 30 s, and extension at 72℃ for 30 s, with a final extension at 72°C for 6 min. |
|  | sca1 | F1→R1 | An initial denaturation for 5 min at 95°C followed by 35 cycles of denaturation at 95℃ for 30 s, annealing at 50℃ for 30 s, and extension at 72℃ for 30 s, with a final extension at 72°C for 6 min. |
|  |  | F2→R2 | An initial denaturation for 5 min at 95°C followed by 35 cycles of denaturation at 95℃ for 30 s, annealing at 55℃ for 30 s, and extension at 72℃ for 30 s, with a final extension at 72°C for 6 min. |
|  | gltA | F1→R1 | An initial denaturation for 5 min at 95°C followed by 35 cycles denaturation at 95℃ for 30 s, annealing at 50℃ for 30 s, and extension at 72℃ for 60 s, with a final extension at 72°C for 6 min. |
|  |  | F2→R2 | An initial denaturation for 5 min at 95°C followed by 35 cycles denaturation at 95℃ for 30 s, annealing at 50℃ for 30 s, and extension at 72℃ for 60 s, with a final extension at 72°C for 6 min. |
