# Supplementary material for: Clinical and historical infection of Tacheng tick virus 2: A retrospective investigation
Source: PLoS Negl Trop Dis. 2024 Jun 13;18(6):e0012168. doi: 10.1371/journal.pntd.0012168 (PMC11175498; doi:10.1371/journal.pntd.0012168)
Supplement: S3 Table — (DOCX) [file pntd.0012168.s006.docx]

**Table S3.** Serological examination of the local herdsmen using ELISA.

| Classification | | Serum samples | No. of seropositivity | | Percentage  (95% confidence interval) | |
| --- | --- | --- | --- | --- | --- | --- |
|  |  |  | IgG | IgM | IgG | IgM |
| Village | Yahu | 199 | 14 | 0 | 7.04 (3.90-11.52) | 0 |
|  | Tuanzhuang | 196 | 4 | 0 | 2.04 (0.56-5.14) | 0 |
|  | Kansuwate | 179 | 45 | 5 | 25.14 (18.97-32.16) | 2.79 (0.91-6.4) |
|  | Basitao | 204 | 20 | 17 | 9.80 (6.09-14.73) | 8.33 (4.93-13.01) |
|  | Beimufangzi | 206 | 11 | 7 | 5.34 (2.7-9.35) | 3.40 (1.38-6.88) |
| Gender | Male | 447 | 34 | 7 | 7.61 (5.32-10.47) | 1.57 (0.63-3.2) |
|  | Female | 537 | 60 | 22 | 11.17 (8.64-14.15) | 4.20 (2.58-6.14) |
| Age | <20 | 16 | 2 | 0 | 12.5 (1.55-38.35) | 0 |
|  | 20~59 | 843 | 70 | 28 | 8.30 (6.53-10.37) | 3.32 (2.22-4.76) |
|  | ≥60 | 125 | 22 | 1 | 17.6 (11.37-25.43) | 0.80 (0.02-4.38) |
| Degree of education | Junior middle school and below | 882 | 84 | 21 | 9.52 (7.67-11.66) | 2.38 (1.48-3.62) |
|  | Senio rmiddle school and above | 102 | 10 | 8 | 9.80 (3.32-14.33) | 7.84 (2.19-12.36) |
| Total | | 984 | 94 | 29 | 9.55 (7.79-11.56) | 2.95 (1.98-4.21) |
