# Supplementary material for: Clinical and historical infection of Tacheng tick virus 2: A retrospective investigation
Source: PLoS Negl Trop Dis. 2024 Jun 13;18(6):e0012168. doi: 10.1371/journal.pntd.0012168 (PMC11175498; doi:10.1371/journal.pntd.0012168)
Supplement: S4 Table — (DOCX) [file pntd.0012168.s007.docx]

**Table S4**. Direct link to access the accession number.

| Accession number | Direct link |
| --- | --- |
| MN395475 | https://www.ncbi.nlm.nih.gov/nuccore/MN395475 |
| MN388782 | https://www.ncbi.nlm.nih.gov/nuccore/MN388782 |
| MK801756 | https://www.ncbi.nlm.nih.gov/nuccore/MK801756 |
| MK820045 | https://www.ncbi.nlm.nih.gov/nuccore/MK820045 |
| MT248267 | https://www.ncbi.nlm.nih.gov/nuccore/MT248267 |
| MT248270 | https://www.ncbi.nlm.nih.gov/nuccore/MT248270 |
| MW725300 | https://www.ncbi.nlm.nih.gov/nuccore/MW725300 |
| MN427915 | https://www.ncbi.nlm.nih.gov/nuccore/MN427915 |
| MT248266 | https://www.ncbi.nlm.nih.gov/nuccore/MT248266 |
| MT248268 | https://www.ncbi.nlm.nih.gov/nuccore/MT248268 |
| MN427913 | https://www.ncbi.nlm.nih.gov/nuccore/MN427913 |
| MT237575 | https://www.ncbi.nlm.nih.gov/nuccore/MT237575 |
| MT219896 | https://www.ncbi.nlm.nih.gov/nuccore/MT219896 |
| MT237574 | https://www.ncbi.nlm.nih.gov/nuccore/MT237574 |
| MT219894 | https://www.ncbi.nlm.nih.gov/nuccore/MT219894 |
